# Supplementary material for: Content-rich biological network constructed by mining PubMed abstracts
Source: BMC Bioinformatics. 2004 Oct 8;5:147. doi: 10.1186/1471-2105-5-147 (PMC528731; doi:10.1186/1471-2105-5-147)
Supplement: Additional File 2 — The original results of the above study (non-essential files are deleted to keep the file size under the limit set by BMC bioinformatics). [file 1471-2105-5-147-S2.bz2 › chilibotAdditionalFile2/dip05/712ID9732274E18049/html/PNP_RNE.html]

 


 **PNP** and **RNE** 
  
Found 60 abstracts in PubMed, retrieved 05.  
 

 What does Google say? 
 PDF only 
| .edu only 

---

**Interactive relationship** (e.g. stimulation, inhibition, etc)

**Stimulatory relationship**- However, transiently increasing intracellular poly A levels has also been shown to stabilize the  **pnp**  and  **rne**  transcripts, leading to increased polynucleotide phosphorylase  [ **PNP** ]  PNPase  [ **PNP** ]  and RNase E  [ **RNE** ]  levels respectively.  Ref: 12207699 Mol Microbiol, 2002
**Neutral relationship**- A Streptomyces coelicolor functional orthologue of Escherichia coli RNase E  [ **RNE** ]  shows shuffling of catalytic and PNPase  [ **PNP** ]  binding domains.  Ref: 12675796 Mol Microbiol, 2003

**Non-interactive relationship** (e.g. studied together, co-existance, homology, etc.)

- Using an E. coli two hybrid system, together with BIAcore apparatus, we investigated the ability of three proteins, polynucleotide phosphorylase  [ **PNP** ]  PNPase  [ **PNP** ] , RhlB RNA helicase, and enolase, a glycolytic protein, to interact physically and functionally independently of RNase E  [ **RNE** ] .  Ref: 12181321 J Biol Chem, 2002
- Furthermore, in the absence of RNase E  [ **RNE** ] , purified RhlB can carry out ATP dependent unwinding of double stranded RNA and consequently modulate degradation of double stranded RNA together with the exonuclease activity of PNPase  [ **PNP** ] .  Ref: 12181321 J Biol Chem, 2002
- Deletion analysis mapped the catalytic domain of RNase ES near its centre and showed that regions located near the RNase ES termini interact with an S. coelicolor homologue of polynucleotide phosphorylase  [ **PNP** ]  PNPase  [ **PNP** ]  a major component of E. coli RNase E  [ **RNE** ]  based degradosomes.  Ref: 12675796 Mol Microbiol, 2003
- Polyadenylation of Escherichia coli transcripts plays an integral role in regulating intracellular levels of polynucleotide phosphorylase  [ **PNP** ]  and RNase E  [ **RNE** ] .  Ref: 12207699 Mol Microbiol, 2002
- These results provide evidence for the first time that both functional and physical interactions of individual degradosome protein components can occur in the absence of RNase E  [ **RNE** ]  and raise the prospect that the RNase E  [ **RNE** ]  independent complexes of RhlB RNA helicase and PNPase  [ **PNP** ] , detected in vivo, may constitute mini machines that assist in the degradation of duplex RNA in structures physically distinct from multicomponent RNA degradosomes.  Ref: 12181321 J Biol Chem, 2002
- Thus, it appears that polyadenylation of transcripts in E. coli serves as a sensing mechanism by which the cell adjusts the levels of both RNase E  [ **RNE** ]  and PNPase  [ **PNP** ] .  Ref: 12207699 Mol Microbiol, 2002
- Here, we show that the half lives of both the  **pnp**  and  **rne**  transcripts are dependent on the intracellular level of polyadenylated transcripts.  Ref: 12207699 Mol Microbiol, 2002
- In addition, experiments using  **pnp**  lacZ and  **rne**  lacZ translational fusions demonstrate that the variations in transcript stability and protein levels arise from alterations in the autoregulation of both genes.  Ref: 12207699 Mol Microbiol, 2002
- DEAD box RhlB RNA helicase physically associates with exoribonuclease PNPase  [ **PNP** ]  to degrade double stranded RNA independent of the degradosome assembling region of RNase E  [ **RNE** ] .  Ref: 12181321 J Biol Chem, 2002
